# DNA Barcoding for Identification of Consumer-Relevant Fungi Sold in New York: A Powerful Tool for Citizen Scientists?

**DOI:** 10.3390/foods7060087

**Published:** 2018-06-08

**Authors:** Emily Jensen-Vargas, Christine Marizzi

**Affiliations:** 1 Francis Lewis High School, New York City Department of Education, Queens, NY 11365, USA; ejensenvargas@gmail.com; 2 DNA Learning Center, Cold Spring Harbor Laboratory, Cold Spring Harbor, NY 11724, USA

**Keywords:** DNA barcoding, citizen science, edible mushrooms, online databases

## Abstract

Although significant progress has been made in our understanding of fungal diversity, identification based on phenotype can be difficult, even for trained experts. Fungi typically have a cryptic nature and can have a similar appearance to distantly related species. Moreover, the appearance of industrially processed mushrooms complicates species identification, as they are often sold sliced and dried. Here we present a small-scale citizen science project, wherein the participants generated and analyzed DNA sequences from fruiting bodies of dried and fresh fungi that were sold for commercial use in New York City supermarkets. We report positive outcomes and the limitations of a youth citizen scientist, aiming to identify dried mushrooms, using established DNA barcoding protocols and exclusively open-access data analysis tools for species identification. Our results indicate that the single-locus nuclear ribosomal internal transcribed spacer (ITS) DNA barcoding approach allowed for identification of only a subset of all of the samples at the species level, although the generated high-quality DNA barcodes were submitted to three different databases. Our results highlight the need for a curated, centralized, and open access ITS reference database that allows rapid third-party annotations for the benefit of both traditional research as well as the emerging citizen science community.

## 1. Introduction

Edible and consumer-relevant fungi are of high economic value and are imported to the United States from as many as fifty different countries [1]. For the year 2007, the value of imported fungi (excluding those that are prepared or preserved by vinegar or acetic acid) totaled more than $289 million [1]. Mushrooms are the spore-producing structures of certain fungi, typically consisting of a stalk and a cap, and in most species, the spores are formed on gills. Mushrooms that are available on the market for human consumption are either harvested in the wild or farm-raised. Reliably identifying foods can be a challenge, especially when they belong to highly speciose and understudied groups like fungi. Fungal taxonomy and morphology is challenging because of their typically cryptic nature and the diversity they exhibit in morphology, ecology, and lifecycles [2,3]. Adding to this difficulty is the appearance of industrially processed mushrooms, which is, in many cases, not clear enough for accurate species identification, as they are often sold sliced and dried [4]. This may contribute to the mislabeling of mushrooms and their products on the market. 

Many foods are commonly mislabeled in order to gain a greater profit. Cheaper products are replaced with expensive ones [5]. Examples of this include the substitution of white tilapia for tuna in sushi [6], and horsemeat instead of beef in spaghetti sauce [5]. Similar fraud has been found within the medicinal raw drug market, where the contamination and substitution of herbal products has been reported [7,8]. Reports of food fraud and medicinal marketplace replacements are met with high interest by the general public [6], and, since citizen scientists could be directly affected by purchasing fraudulent products, they enjoy investigating these topics. This study was inspired by a previous report that the molecular identification of fungi in a commercial packet of dried Chinese porcini mushrooms, purchased in a London supermarket, yielded three novel species that had never formally been described by science [9]. In this study, we were interested to see if a 14 year old New York City (NYC) high school student could mirror this report in New York. The youth citizen scientist followed a simplified workflow to identify the species of fungi that were sold in New York, including fresh samples and two commercial packets of dried mushrooms that were purchased in supermarkets. Moreover, the participating youth citizen scientist was particularly interested in potentially uncovering fraudulent marketplace replacements. 

Citizen science, the active involvement of non-professional scientists in research, is experiencing an upsurge of interest. Citizens joining as active contributors in scientific research are increasingly addressing both societally relevant and fundamental research questions [10]. In addition, citizen science projects have been incorporated into multiple science education settings globally, ranging from secondary to higher education, as well as teacher trainings. Particularly compelling are the projects that are scalable and adaptable to multiple situations, are conceptually and technically straightforward, and are using online open-access tools that allow participants to contribute high-quality data to science. In science education, DNA barcoding projects provide ideal ways to stimulate independent student thinking across different levels of biological organization, by linking molecular genetics to ecology and evolution. DNA barcoding is an established biotechnological tool that uses short, standardized genetic markers to objectively identify the species of almost every specimen. DNA barcoding also integrates different methods of scientific investigation—from in vivo observations to in vitro biochemistry to in silico bioinformatics. The core lab and sequence analysis work can be mastered in a relatively short time, allowing students and citizen scientists to reach a satisfying research endpoint within a short period of time. DNA barcoding has been widely used to identify fungi taxonomically [11], although with reportedly varying success [12,13,14]. Animal and plant barcode regions target the variable 5′ portion of cytochrome oxidase 1 (*COI*), and the large subunit of RuBisCo (*rbcL*) and maturase K (*matK*) as core-barcodes, respectively [15,16,17]. For fungi, the nuclear ribosomal internal transcribed spacer (ITS) has been selected as the standardized DNA barcode by the International Fungal Barcoding Consortium [3,13,18,19,20].

Here, we report the positive outcomes and limitations of a youth citizen scientist aiming to identify fresh and dried mushrooms, using established DNA barcoding protocols and fungal specific primers. We were interested whether a youth citizen scientist, who was new to molecular genetics, could identify all of the fungal species in a bag of sliced and dried mushrooms. Citizen scientists often conduct projects within a limited amount of time and support, in terms of lab access and funding. Creating a real-life scenario, the youth citizen scientist was given lab access for a defined period of time as well as the funds to generate up to 30 DNA barcodes. However, the student was responsible for the overall project design and sample collection; therefore, the sample size was limited to what could have been easily obtained by the citizen scientist. As the quality of data that are generated by citizen scientists is generally of concern for the scientific community [21], we verified the accuracy of our generated fungi barcodes through submission to three online and open-access search tools that allowed species identification using nucleotide sequences. 

## 2. Materials and Methods 

We followed the guidelines and protocols that were published by the Cold Spring Harbor Laboratory’s DNA Learning Centers’ DNA Barcoding 101 website [22], with minor modifications, as outlined below. 

### 2.1. Study Limitations in Time, Funding, and Lab Access

All of the laboratory work was conducted after school and on select Saturdays at the Harlem DNA Lab in NYC, NY, USA. The lab access was limited to five months, for three hours per week. All of the materials and reagents that were needed to generate up to 30 DNA barcodes were provided, except for the funds to cover the costs that were associated with obtaining the samples. Sample collection was the responsibility of the youth citizen scientist.

### 2.2. Sample Collection

Dried and fresh fungi samples were collected from local supermarkets in two NYC boroughs (Manhattan and Queens) in February 2017. The dried samples were obtained from two bags of sliced and dried fungi, which were sold as edible “Dried Stir Fry Mushroom Blend” and “Dried black mushroom”. The “Dried Stir Fry Mushroom Blend” was distributed by an American vendor, based in Pennsylvania, United States. The bag’s contents were described as a medley of five different mushrooms, listed as “Shiitake, Shredded Wood Ear, Sliced Button, Cloud Ear, and Oyster”. The package indicated a 28 g net weight and lot number L981553, but had no country of origin. The second bag of “Dried black mushroom” was of Chinese origin, packaged in Korea, and distributed by a Pan Asian food importer, based in Maryland, United States. The label indicated a 56.7 g net weight with no lot number. We referred to this sample as “Korean Black” throughout the manuscript. The Latin binominal names were not displayed on either of the package labels. The fresh samples were purchased at local supermarkets, yet only two varieties were available at the time of sampling, Yellow Foot and White Button. The samples were visually sorted by phenotype, and were documented and vouchered. The samples were then potentially identified by morphology. While the fresh samples were easy to identify, many of the dried samples were broken down into smaller pieces and therefore lacked the key morphological characteristics that could have been used for identification. One representative of each of the visually sorted samples was removed arbitrarily, and all of these samples (*n* = 10) were further analyzed via DNA barcoding. 

### 2.3. Generating DNA Barcodes

The DNA was extracted from approximately 0.1 g of the specimen, using a standard silica- based DNA extraction method (as recommended by the DNA Barcoding 101 webpage [22]) with one minor modification, which was that for each sample, a small piece was removed, placed in a sterile 1.5 mL microcentrifuge tube, and pre-soaked in dH_2_O, before being ground in a 300 μL lysis buffer (6 M guanidine hydrochloride solution (Sigma-Aldrich, St. Louis, MO, USA)) with a sterile pestle. The extracted DNA was amplified by polymerase chain reaction (PCR), using fungi-specific ITS primers (ITS1F/ITS4, as described by the authors of [13]) with attached M13F and M13R sequencing ‘tails’ (ITS1F-M13 5′-**TGTAAAACGACGGCCAGT**CCGTAGGTGAACCTGCGG-3′ and ITS4R-M13 5′-**CAGGAAACAGCTATGAC**TCCTCCGCTTATTGATATGC-3′, the incorporated M13 sequences for sequencing were in bold). The PCR was performed using an NEB Taq 2X master mix (New England Biolabs, New York, NY, USA), following the thermocycler parameters that were outlined by the DNA Barcoding 101 website [22]. Negative and positive controls were included to ensure that the PCR amplicons were neither contaminations nor artifacts. Then, the amplified DNA was stained with SYBR^TM^ Safe DNA Gel Stain (Invitrogen, Carlsbad, CA, USA) and was confirmed by a 2% agarose gel electrophoresis. The gels were viewed on a UV transillumination plate (FOTODYNE, Hartland, WI, USA, emitting UV light at 300 nm) and photographed using a digital camera and the FOTODYNE photodocumentary system (FOTO/Analyst Express Zoom Lens Systems and PC Image software Version 5.00, Hartland, WI, USA). The positive amplicons were sent to the GENEWIZ INC South Plainfield facility in New Jersey for bi-directional sequencing, using universal M13F and M13R primers [23]. 

### 2.4. In Silico Species Identification

The open-access DNA Subway software interface [24] was used to analyze the DNA sequences, using the default settings [25,26]. The youth citizen scientist was trained in DNA Subway. ‘Riding’ the ‘Blue Line’ in the DNA Subway, we first uploaded our raw sequencing data, trimmed them in bulk, and then viewed and manually edited the forward and reverse sequences to build a clean consensus sequence for every sample. If the forward and reverse reads could not be combined to produce a consensus sequence, as a result of a low quality of one of them, only the readable barcode sequence was included into the subsequent steps. Then, we performed a Basic Local Alignment Search Tool (BLAST [27]) nucleotide search (BLASTn) to identify any close matches to our sequences in the local sequence database, which was built into DNA Subway. Each sample was potentially ‘identified’ based on the highest max score/bit score. For the sequences with nucleotide ambiguities, the reads in both directions were checked for quality and substantial overlap in the consensus sequence and the proper reading frames. These sequences were flagged for subsequent steps in the analysis, as they would have needed to have been excluded as a result of low quality, if the DNA barcode standards (sequence of at least 500 bp in length with low nucleotide ambiguities) were not met. To independently verify species identifications through DNA Subway’s local database, the sample consensus sequences were downloaded in FASTA format and were submitted directly to the National Center for Biotechnology Information (NCBI)-GenBank BLAST [27] and User-friendly Nordic ITS Ectomycorrhiza (UNITE) Ver. 7.1 [28], an open-access, curated fungi-specific database [29,30]. If these additional searches returned identical identifications, at least to the genus level, we considered the samples identified. For identifications that were based on web BLAST we only referred to the published sequences from Mycological studies. To generate a visual barcode that was easy to read by citizen scientists, our newly generated consensus sequences were aligned with authenticated published sequences (see supplementary materials, Table S1 for selected GenBank accession numbers), using the Multiple Sequence Alignment program, MUSCLE (Version 3.8.31) [31]. Then, a phylogenetic tree was constructed using the PHILYP multiple likelihood (ML) method, using the Phylogeny Inference Package (Version 3.69) [32]. Because a scale bar indicating sequence divergence was not provided by the output of this program, this tree was not shown here. The same set of sequences was aligned using the Clustal Omega Multiple Sequence Alignment (MSA) server, which was maintained by the European Molecular Biology Laboratory’s European Bioinformatics Institute (EMBL-EBI). The alignment was carefully screened for indels and larger mutations, and a second phylogenetic tree was then generated using the maximum likelihood method in Mega7. Visualization was done using FigTree v1.3.1 (Rambaut group, University of Edinburgh, Edinburgh, UK [33]) and was rooted to an *Aspergillus* sequence as an outgroup, to show the relative diversity among the other fungi. The trees were constructed by the youth citizen scientist to look at the relative relatedness of the obtained sequences only, and did not represent an in-depth phylogenetic analysis to confirm the taxonomic identity.

## 3. Results

In this study, 10 mushroom samples were purchased from 3 locations in Manhattan and Queens, New York. Of these, 30% percent were fresh samples and 70% were dried samples (Table 1). 

The extracted DNA of all of the samples underwent PCR twice, and we successfully amplified 90% of the samples (9/10) for the ITS barcode region. The amplicons were then submitted for bidirectional sequencing. 

For one sample (sample S3), we were not able to obtain a DNA sequence because of unsuccessful PCR amplification, in spite of several attempts (Table 1). This may have been as a result of the potentially high degradation of the sample’s DNA because of the heat treatment prior to packaging—sample S3 appeared very hard and dry. Once the DNA was degraded into short fragments, generating ITS barcodes may have been difficult, as the primer binding sites may have been degraded [34,35]. Moreover, secondary metabolites could inhibit PCR by decreasing the Taq-polymerase’s activity, and our simple DNA extraction method may have been unsuccessful in removing these metabolites. Nevertheless, we did not attempt another DNA extraction using a commercially available kit, as barcodes could be obtained from all of the other samples. 

Notably, sample S2 (potentially identified as Shiitake mushroom) only yielded a single sequencing read (reverse direction) in the first sequencing attempt, which, surprisingly, was sufficient to identify this sample with a 99% sequence identity match on GenBank. However, we repeated the sequencing and were able to build a consensus sequence that matched an authenticated published GenBank entry by 99%. Samples S4 and S6 (both potentially identified as Oyster mushroom) produced sequencing reads with less overlap compared with the other samples, increasing the risk for potential sequencing errors that could not be edited in consensus with the second strand; therefore, both of the sequences were flagged as ‘low quality’ for further analyses. The remaining samples produced high-quality barcodes of at least 500 bp in length, with low nucleotide ambiguities, and therefore met the DNA barcode standards (GenBank accession numbers MH394708–MH394714). 

When a sequence was obtained, we were able to determine the fungal identity of all of the samples through DNA Subway, GenBank BLASTn direct comparison, and UNITE Ver. 7.1 serial BLAST search (Table 1 and Table 2, Table S1 in supplementary materials). Because DNA Subway’s local nucleotide database allowed a rapid but limited search (it included NCBI sequences < 20 kb and longer than 400 bp, matching certain search terms, and was updated monthly) we additionally searched the curated UNITE database. However, we found that the majority (55.6%) of all of the samples were potentially identified only to the genus level, but not to the exact species level through DNA Subway and UNITE (Table 2). For all of the sequences, the top BLAST result was determined using the highest percent identity score. In the case of a tie between two identity scores, the lowest e-value was used as a deciding factor. If there were identical e-values and identity scores for multiple species, the individuals were only identified to the genus level so as to avoid any incorrect taxonomic identifications. Taking the results of all three of the searches in account, we found that samples S1, S2, S5, and S7 were labeled correctly at both the species and genus level. Samples S4, S6, S8, S9, and S10 were labeled at the correct genus level, but we were unable to resolve them down to the species level, as there were minor discrepancies between the databases (see Table 2). In addition, samples S4 and S6 returned top matches in the range of 84% and 93% because of their poor sequence quality, but it was possible to potentially identify the genus. The careful selection of published GenBank reference sequences when performing GenBank BLASTn searches allowed us to identify seven out of nine samples (77.8%) up to the species level (in supplementary materials, Table S1). 

**Figure 1:**
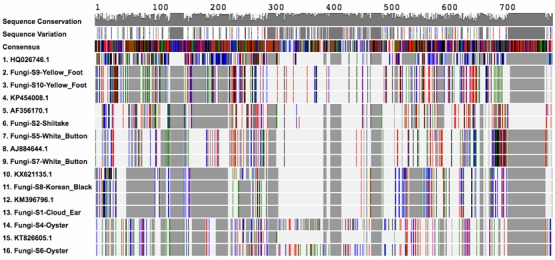
Sample consensus sequences (S1–S10) aligned with corresponding Basic Local Alignment Search Tool nucleotide search (BLASTn) sequences hits and one *Aspergillus fumigatus* sequence, using the MUSCLE multiple alignment software embedded in DNA Subway to create a visual DNA barcode. Sequence lengths are indicated as numbers on the top, and the sequence conservation and variation values are indicated as grey bars in the top three panels. Nucleotides are color coded with green representing A, red representing T, black repenting G, and blue representing C. The columns are matches (or mismatches) at a single nucleotide position across all of the sequences in comparison to the consensus sequence. This ‘Alignment Viewer’ allows citizen scientists to quickly screen for samples generating similar or identical barcodes, indicating that they are closely related or the same species.

**Figure 2:**
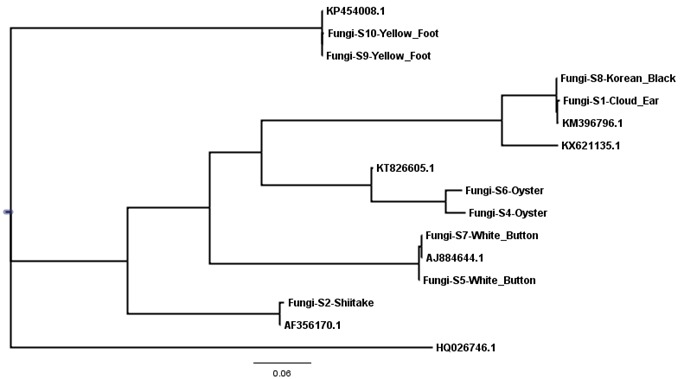
Phylogenetic tree of generated DNA barcodes and closest matching GenBank entries, based on internal transcribed spacer (ITS) sequence diversity. One *Aspergillus fumigatus* sequence, derived from GenBank (accession number HQ026746.1), was added to the sample data set and selected as an outgroup.

A visual barcode and phylogeny for all of fungi samples is depicted in Figure 1 and Figure 2. The Korean Black (S8) and Cloud Ear (S1) sequences clustered with *Auricularia heimuer,* suggesting that these species were likely identical, or that the ITS region may not have had enough variation to differentiate between these species genetically. Both of the Yellow Foot samples (S9 and S10) clustered together with *Craterellus tubaeformis*, and the two Oyster mushroom samples (S4 and S6) clustered with *Pleurotus* sp. In addition, the White Button samples (S5 and S7) clustered with *Agaricus bisporus,* suggesting a correct identification though DNA barcoding. 

## 4. Discussion

Recently, species identification by DNA barcoding has gained popularity in the global citizen scientist community, and several inexpensive lab protocols and computational infrastructures were developed to support this approach. This development was not surprising, as DNA barcoding offered an inquiry-driven format to introduce participants to key biological ideas—including genetics, molecular structure, bioinformatics, ecology, biodiversity, and the impacts of human activity. DNA barcoding followed a relatively straightforward workflow, using minimal equipment that was available at many high schools, colleges, or community labs. The wetlab portion could be completed within a couple of hours using homemade, no-caustic reagents and intuitive, open-access software tools, such as the DNA Subway supported streamlined data analysis. In most cases, citizen scientists could complete every step by themselves, except for the sequencing, which may have been performed by a scientific collaborator or, as in our case, a commercial facility. DNA Subway supported the direct export of high-quality and novel DNA barcodes that had not been published previously at GenBank, thereby making them available for other researchers.

In this study, we were interested whether a youth citizen scientist who was new to molecular genetics could identify all of the fungi species in two bags of sliced and dried mushrooms. Although we worked with a very limited samples set, our results clearly showed that DNA barcoding was a powerful tool for citizen scientists to identify the dried mushroom samples that may have exhibited an unclear phenotype. Uncovering potential food fraud or identifying a new species was the major draw for the student to this project. However, all of the samples matched the existing database entries and there was no case of mislabeling in our 10 tested samples. Within our limited sample set, 22.2% of the samples were potentially identified to the genus level and 77.8% to the species level. While this showed the potential of the chosen DNA barcoding workflow for species identification, these results would have needed to be tested within a much larger sample set. We also found that for a more rigorous sequence analysis, DNA Subway needed to be supplemented by web-based BLAST sequence comparison and phylogenetic analyses, which would output advanced node/clade support. This became apparent as sample S8 (Korean Black) matched *Auricularia heimuer* as well as *Auricularia auricula-judae* with 100% on the DNA Subway local database, and we found the same ambiguity on UNITE. The taxonomic identification of S8 based on the DNA Subway or UNITE BLAST searches would have supported a resolution to the genus level only—*Auricularia* sp. A direct GenBank BLAST search and selection of an authenticated reference sequence, however, supported the identification as *Auricularia heimuer* (99% match to KM396796; see supplementary materials, Figure S1). Additional markers like the intergenetic spacer (IGS) region could have been added to further discriminate between the *Auricularia* species in future citizen science projects [36,37]. 

We did not use the established Barcode of Life Data System (BOLD) identification system [38], as we expected that their employed BLAST algorithm for ITS sequences, instead of the standard BOLD identification engine, would not have returned any supplementary identification. In addition, it was not recommended to submit queries to BOLD at the moment, because there were too few ITS records on BOLD to return a successful match. A UNITE Ver. 7.1 serial BLAST search was utilized in addition to NCBI-GenBank BLASTn, because it was found that 27% of the fungal ITS sequences that were entered into GenBank were inadequately identified and that 20% were incorrectly labeled [39,40]. UNITE provided the identification of the ITS sequence as a species hypothesis, based on sequence similarity, and we found that, while it allowed for a more reliable identification of fungal sequences, the taxonomic identification still needed to be verified through alignment with the authenticated GenBank sequences. Online searches led to the recently launched Faces of Fungi website [41,42], which announced easy deposition of taxonomic data plus phenotypic details and other metadata to enhance current taxonomic understanding. Future studies would benefit greatly from such a curated, centralized, and open access ITS reference database that would allow annotation in a more rapid manner and could serve as a solid foundation for the traditional research as well as the emerging citizen science community. 

## 5. Conclusions

In a time where the number of traditional taxonomists, including fungal taxonomists, is on the decline, citizen scientists can help to rapidly fill this gap. However, the limitations in terms of budget, time, and laboratory space are a significant problem for citizen science projects, specifically if they involve youth citizen scientists, like high school students. Firstly, it would have been beneficial if the costs that were associated with obtaining samples could have been included into the project budget so as to increase the sample set. Alternatively, samples may have been collected in advance and provided to the citizen scientists for their own, potentially small-scale, projects and the generated data could then be reported back and incorporated into larger targeted and curated projects. Secondly, the youth citizen scientist had very limited availability to spend time in the lab to complete the project because of other after-school commitments. The youth citizen scientist also underestimated the time that was needed to finish all of the steps of the project. While the time to complete a DNA barcoding project using an established workflow and with a limited amount of samples seemed to be a relatively small commitment for a professional scientist, this could pose a significant challenge for participants new to science. In addition to weekly three hour long sessions after school, we would suggest adding more sessions. If the time commitment of the citizen scientist volunteer allowed for it, longer lab hours would have enabled the completion of more steps in the same session. Another challenge remains the in depth-analysis of obtained DNA sequences. Reliable identification of species through DNA barcoding greatly relied on the representation of relevant taxa in the reference database. We recommend supplementing pedagogical open-access software, such as DNA Subway, with web-based BLAST sequence comparison and additional phylogenetic analyses.

This study was inspired by a youth citizen scientist who aimed to uncover potential food mislabeling in commercially available fungi. The fact that we did not find food mislabeling with our limited samples does not mean that food fraud is not conducted. However, we hope that this study inspires many more citizen scientists to help with expanding the sample set. Accessible DNA barcoding can offer an effective method to help provide more accurate ingredient labels to consumers, thereby improving food safety. This method also has shown great potential to give back control to the consumers. We conclude that DNA barcoding empowers citizen scientists to identify fungi species and make discoveries that are meaningful both personally and for the scientific community.

## Figures and Tables

**Table 1:** List of fungi sampled in this study for internal transcribed spacer (ITS) barcoding. The taxonomic identifications (ID) of the samples based on the User-friendly Nordic ITS Ectomycorrhiza Database (UNITE) serial Basic Local Alignment Search Tool (BLAST) search for ITS barcodes and common names are indicated. Samples S1–S6 were derived from the bag of “Dried Stir Fry Mushroom Blend”. Sample S8 was derived from the bag of “Dried black mushrooms.” Scale bar indicates approximately 1 cm. Scale bar for samples S9 and S10 is not available.

Sample	Date Purchased	Location of Purchase	Package ID	Picture	Barcode ID Based on UNITE Serial BLAST Search
S1	2 February 2017	Supermarket, Queens, NY	Cloud Ear (dried)	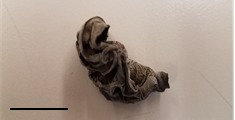	*Auricularia heimuer* (Cloud Ear)
S2	2 February 2017	Supermarket, Queens, NY	Shiitake (dried)	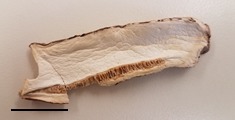	*Lentinula edodes* (Shiitake mushroom)
S3	2 February 2017	Supermarket, Queens, NY	Wood Ear (dried)	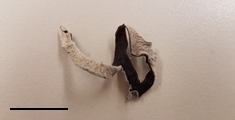	No sequence
S4	2 February 2017	Supermarket, Queens, NY	Oyster mushroom (dried)	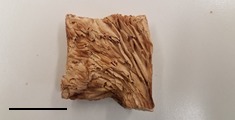	*Pleurotus* sp. (Oyster mushroom)
S5	2 February 2017	Supermarket, Queens, NY	White Button (dried)	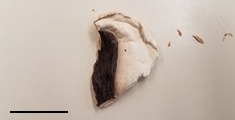	*Agaricus bisporus* (White Button)
S6	2 February 2017	Supermarket, Queens, NY	Unknown (dried)	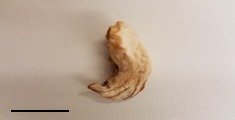	*Pleurotus* sp. (Oyster mushroom)
S7	2 February 2017	Supermarket, Queens, NY	White Button (fresh)	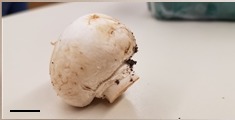	*Agaricus bisporus* (White Button)
S8	2 February 2017	Supermarket, Queens, NY	Korean Black (dried)	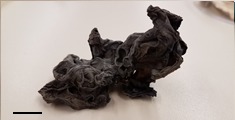	*Auricularia* sp. (Wood Ear)
S9	24 February 2017	Supermarket, Manhattan, NY	Yellow Foot (fresh)	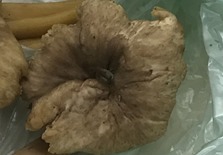	*Craterellus* sp. (Chanterelle)
S10	24 February 2017	Supermarket, Manhattan, NY	Yellow Foot (fresh)	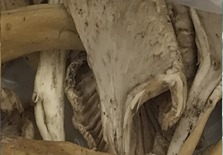	*Craterellus* sp. (Chanterelle)

**Table 2:** Species identification based on internal transcribed spacer (ITS) DNA barcoding from all of the samples analyzed (*n* = 10). Each consensus sequence of samples S1–S10 was subjected to three search tools to verify identity (ID), namely: 1) DNA Subway; 2) direct submission to Basic Local Alignment Search Tool (BLAST) search in GenBank; and 3) a serial BLAST search in the curated fungal taxonomic reference database User-friendly Nordic ITS Ectomycorrhiza Database (UNITE). * indicates low-quality sequences, in both instances of the Oyster mushroom samples. N/A: not available.

Sample	Fungal sp. Per Label	DNA Subway		BLAST, GenBank		UNITE	
		Barcode ID	% Match	Barcode ID	% Match	Barcode ID	% Match
S1	Cloud Ear	*Auricularia heimuer*	100	*Auricularia heimuer*	99	*Auricularia heimuer*	100
S2	Shiitake mushroom	*Lentinula edodes*	100	*Lentinula edodes*	99	*Lentinula edodes*	100
S3	Wood Ear	N/A	N/A	N/A	N/A	N/A	N/A
S4 *	Oyster mushroom	*Pleurotus ostreatus*	84	*Pleurotus* sp.	93	*Pleurotus* sp.	85
S5	White Button	*Agaricus bisporus*	100	*Agaricus bisporus*	99	*Agaricus bisporus*	100
S6 *	Oyster mushroom	*Pleurotus* sp.	90	*Pleurotus* sp.	91	*Pleurotus* sp.	91
S7	White Button (fresh)	*Agaricus bisporus*	100	*Agaricus bisporus*	100	*Agaricus bisporus*	100
S8	Korean Black	*Auricularia* sp.	100	*Auricularia heimuer*	100	*Auricularia* sp.	100
S9	Yellow Foot (fresh)	*Craterellus tubaeformis*	100	*Craterellus tubaeformis*	100	*Craterellus* sp.	100
S10	Yellow Foot (fresh)	*Craterellus tubaeformis*	100	*Craterellus tubaeformis*	100	*Craterellus* sp.	100

## Supplementary Materials

The following are available online at http://www.mdpi.com/2304-8158/7/6/87/s1, Table S1: Identification and consensus sequence information of all samples. Figure S1: A Clustal Omega alignment of samples S1 (Cloud Ear) and S8 (Korean Black), and two GenBank reference sequences (KM396796 (*Auricularia heimuer*) and KX621135 (*Auricularia auricula-judae*)).
