# Supplementary figures and images for: DNA Barcoding for Identification of Consumer-Relevant Fungi Sold in New York: A Powerful Tool for Citizen Scientists?
Source: Foods. 2018 Jun 8;7(6):87. doi: 10.3390/foods7060087 (PMC6025134; doi:10.3390/foods7060087)

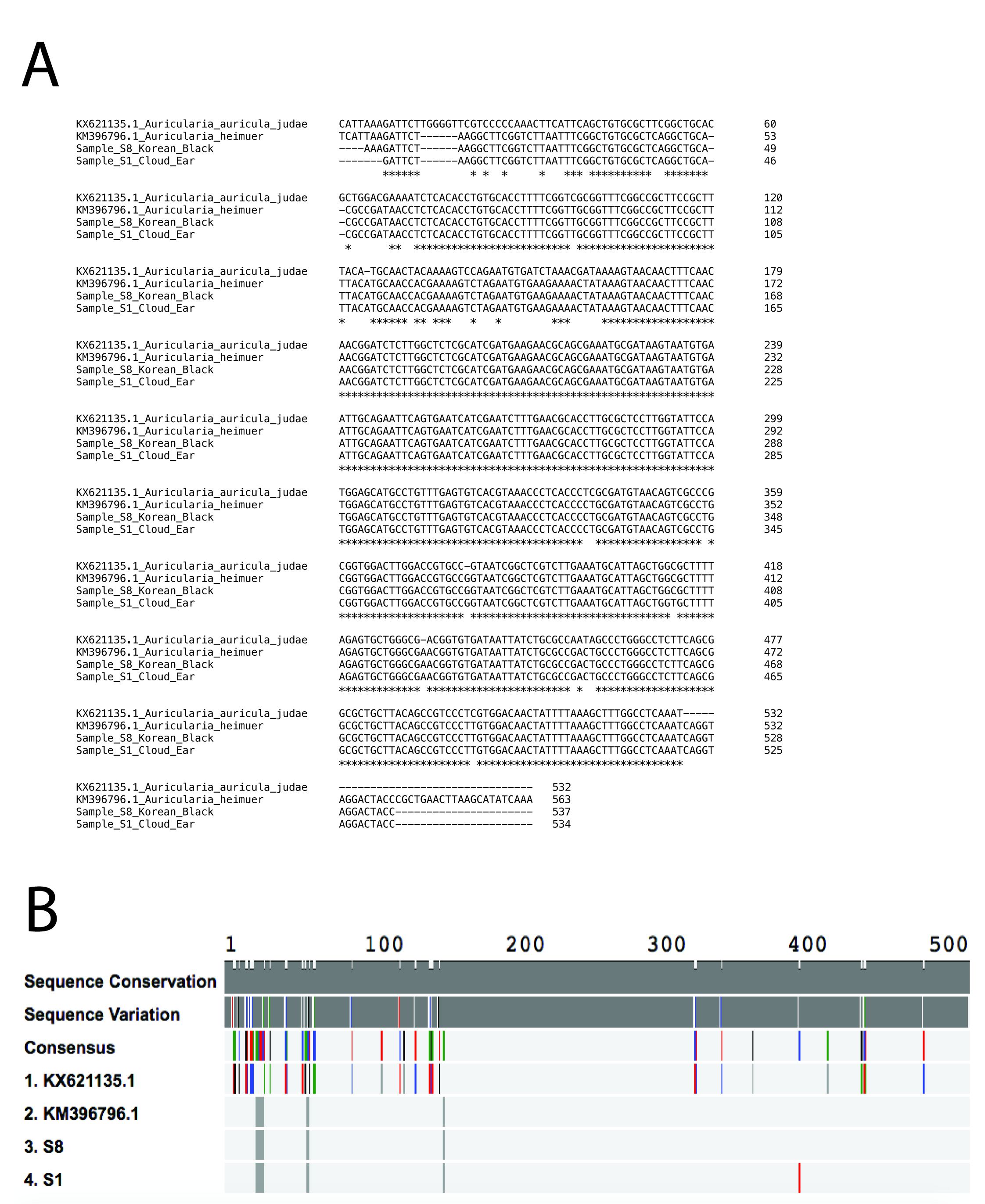

Supplement: Supplementary file 1 [file foods-07-00087-s001.zip › Foods-303306-supplementary /Supplementary_figure_S1.tif]
